# Binge ethanol exposure during adolescence leads to a persistent loss of neurogenesis in the dorsal and ventral hippocampus that is associated with impaired adult cognitive functioning

**DOI:** 10.3389/fnins.2015.00035

**Published:** 2015-02-12

**Authors:** Ryan P. Vetreno, Fulton T. Crews

**Affiliations:** Department of Psychiatry, Bowles Center for Alcohol Studies, School of Medicine, University of North CarolinaChapel Hill, NC, USA

**Keywords:** alcohol, adolescence, object recognition memory, innate immune, hippocampus

## Abstract

Adolescence is a developmental period that coincides with the maturation of adult cognitive faculties. Binge drinking is common during adolescence and can impact brain maturation. Using a rodent model of adolescent intermittent ethanol (AIE; 5.0 g/kg, i.g., 20% EtOH w/v; 2 days on/2 days off from postnatal day [P]25 to P55), we discovered that AIE treatment reduced neurogenesis (i.e., doublecortin-immunoreactive [DCX + IR] cells) in both the dorsal and ventral hippocampal dentate gyrus of late adolescent (P56) male Wistar rats that persisted during abstinence into adulthood (P220). This reduction in neurogenesis was accompanied by a concomitant reduction in proliferating cells (Ki-67) and an increase in cell death (cleaved caspase-3). In the hippocampus, AIE treatment induced a long-term upregulation of neuroimmune genes, including Toll-like receptor 4 (TLR4) and its endogenous agonist high-mobility group box 1 as well as several proinflammatory signaling molecules. Administration of lipopolysaccharide, a gram-negative endotoxin agonist at TLR4, to young adult rats (P70) produced a similar reduction of DCX + IR cells that was observed in AIE-treated animals. Behaviorally, AIE treatment impaired object recognition on the novel object recognition task when assessed from P163 to P165. Interestingly, object recognition memory was positively correlated with DCX + IR in both the dorsal and ventral hippocampal dentate gyrus while latency to enter the center of the apparatus was negatively correlated with DCX + IR in the ventral dentate gyrus. Together, these data reveal that adolescent binge ethanol exposure persistently inhibits neurogenesis throughout the hippocampus, possibly through neuroimmune mechanisms, which might contribute to altered adult cognitive and emotive function.

## Introduction

Adolescence is a conserved neurodevelopmental period in humans and other mammalian species characterized by a surge in cortical and limbic system plasticity that parallels increased social interactions and engagement in risky behaviors (i.e., novelty- and sensation-seeking). The adolescent-typical increase in neuroplasticity is particularly evident in the limbic hippocampal formation where elevated levels of neurogenesis are observed in the adolescent dentate gyrus compared to adults (He and Crews, 2007). Neurogenesis is a process involving the generation and functional integration of newborn neurons into existing brain circuitry (Zhao et al., 2006, 2008), and is highly conserved across mammalian species (Kuhn et al., 1996; Gould et al., 1999b), including humans (Eriksson et al., 1998). Within the hippocampus, neurogenesis is restricted to the subgranular zone of the hippocampal dentate gyrus, which contains a mitotically active microenvironment that permits neurogenesis to occur throughout life (Abrous et al., 2005). It is a highly dynamic process modulated by many intrinsic and extrinsic factors, including neurotransmitters [e.g., acetylcholine (Cooper-Kuhn et al., 2004)], environmental enrichment (Cotman and Berchtold, 2002), pathological insults (Richardson et al., 2007), and drugs of abuse (He et al., 2005). The heightened vulnerability of the neurogenic process to outside influences is particularly relevant during adolescence as this is a period when individuals begin experimentation with drugs and alcohol. Although adolescent binge ethanol exposure has been shown to cause a persistent loss of neurogenesis in primates (Taffe et al., 2010) that in rats is unique to adolescence (Broadwater et al., 2014), the long-term consequences of alcohol exposure during adolescence on adult neurogenesis in the dorsal and ventral hippocampal dentate gyrus is unknown.

Adolescent risk-taking and sensation-novelty seeking coincide with increased experimentation with alcohol and other drugs of abuse (Windle et al., 2008). Binge drinking, defined as the consumption of 5 or more consecutive alcoholic beverages in a 2-h period, is common during adolescence as 5% of 8th grade, 14% of 10th grade, and 22% of 12th grade individuals report engaging in binge drinking over the past 2 weeks (Johnston et al., 2013). This heavy drinking pattern continues through the college years as 44% of students report binge drinking every 2 weeks, and 19% report more than 3 binge drinking episodes per week (Wechsler et al., 1995; O'Malley et al., 1998). Unfortunately, an earlier age of drinking onset (i.e., 11–14 years of age) is associated with an escalated risk of developing an alcohol use disorder later in life (Dewit et al., 2000). Routine binge drinking might lead to long-term changes in hippocampal neurobiology due to the heightened neural plasticity and structural development that characterizes the adolescent brain (Crews et al., 2007). The adolescent brain is particularly sensitive to ethanol-induced inhibition of neurogenesis (Crews et al., 2006b) which might contribute to deficits in cognitive functioning. Indeed, reductions of neurogenesis in the dorsal hippocampal dentate gyrus have been reported in adolescent binge drinking models (Crews et al., 2006b; Ehlers et al., 2013; Broadwater et al., 2014) as well as impairments in hippocampal-dependent memory (White and Swartzwelder, 2004). However, the persistent effects of adolescent binge ethanol exposure on neurogenesis in the dorsal and ventral hippocampus are unknown.

Although the mechanism underlying ethanol-induced reductions of neurogenesis remain to be elucidated, there is accumulating evidence implicating the neuroimmune system in contributing to altered neurogenesis in pathological brain diseases (Whitney et al., 2009; Mathieu et al., 2010). Indeed, in a chronic ethanol administration model, treatment with butylated hydroxytoluene, a potent antioxidant, prevented the ethanol-induced loss of neurogenesis and reduced activation of NF-κB in the hippocampus (Crews et al., 2006a). We report here for the first time that adolescent intermittent ethanol (AIE) treatment reduces neurogenesis (i.e., DCX + IR) in both the dorsal and ventral hippocampal dentate gyrus of late adolescent rats (P56) that persists into adulthood (P220). The observed reduction of neurogenesis was accompanied by decreased cellular proliferation, as measured by Ki-67 immunoreactivity, and increased expression of cleaved caspase-3 immunopositive cells in the dentate gyrus. Further, we discovered that AIE caused long-term upregulation of neuroimmune genes in the young adult hippocampus, and that lipopolysaccharide-induced upregulation of neuroimmune genes mimics the effects of adolescent binge drinking on neurogenesis. Finally, AIE treatment led to impaired object recognition memory and altered anxiety-like behavior in adulthood that was correlated with hippocampal neurogenesis. Together, these data suggest that adolescent binge ethanol treatment leads to persistent reductions of neurogenesis in both the dorsal and ventral hippocampal dentate gyrus that might involve neuroimmune processes and contribute to cognitive and emotive dysfunction in adulthood.

## Materials and methods

### Animals

Young time-mated pregnant female Wistar rats (embryonic day 17; Harlan Sprague-Dawley, Indianapolis, IN) were acclimated to our animal facility prior to birthing at the University of North Carolina at Chapel Hill. On postnatal day (P)1 (24 h after birth), litters were culled to 10 pups and housed with their mothers in standard clear plastic tubs with shavings until group housing with same-sex littermates at the time of weaning on P21. All animals were housed in a temperature- (20°C) and humidity-controlled vivarium on a 12 h/12 h light/dark cycle (light onset at 07:00 h), and provided *ad libitum* access to food and water. Experimental procedures were approved by the IACUC of the University of North Carolina at Chapel Hill, and conducted in accordance with NIH regulations for the care and use of animals in research.

### Adolescent intermittent ethanol (AIE) treatment

**Figure 1:**
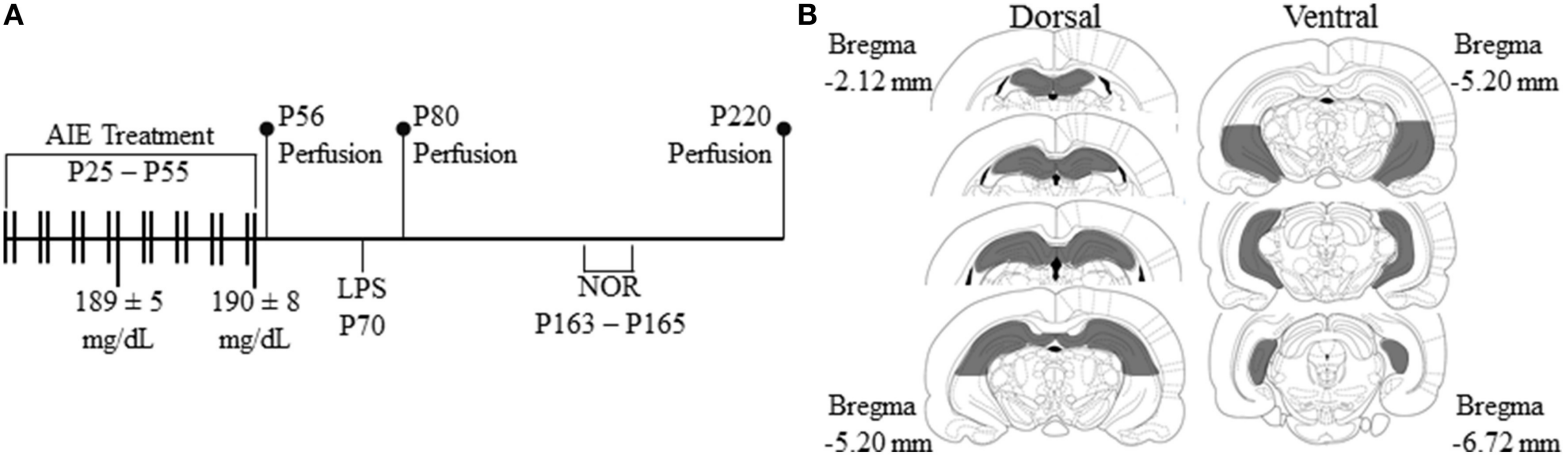
**Graphical representation of the adolescent intermittent ethanol (AIE) exposure protocol and experimental design. (A)** Rats were treated with either ethanol (5.0 g/kg, 20% ethanol w/v, i.g.) or a comparable volume of water on a 2-day on/2-day off administration schedule from postnatal day (P) 25 to P55. Blood ethanol concentrations (BECs) were assessed 1 h after ethanol exposure on P38 and P54 (mg/dL). Late adolescent rats were sacrificed on P56 (24 h post-AIE treatment). Subjects in the early adulthood group were sacrificed on P80 (25 days post-AIE treatment). A subset of subjects in the early adulthood group were treated with lipopolysaccharide (LPS; 1.0 mg/kg, i.p.) on P70 and sacrificed on P80. Subjects in the adulthood group were behaviorally tested on the novel object recognition memory task from P163 to P165, and were sacrificed on P220 (165 days post-AIE treatment). **(B)** Depicted are coronal sections of the dorsal and ventral hippocampus between Bregma –2.12 mm and –6.72 mm according to the atlas of Paxinos and Watson (1998) used for quantitative analysis of doublecortin-immunopositive cells.

On P21, male Wistar rats were randomly assigned to either (i) AIE or (ii) water control (CON) groups. From P25 to P55, AIE animals received a single daily intragastric (i.g.) administration of ethanol (5.0 g/kg, 20% ethanol w/v) on a 2-day on/2-day off schedule and CON subjects received comparable volumes. Tail blood was collected to assess blood ethanol content (BEC) 1 h after ethanol administration as we previously found that BECs in the adolescent rat peak at approximately 60 min after i.p. ethanol administration (Crews et al., 2006b). Further, BECs were assessed at the midpoint of AIE treatment (P38) and again at the conclusion of AIE treatment (P54), and were quantitated using a GM7 Analyzer (Analox; London, UK). On P38 and P54, mean BECs (± SEM) were 189 ± 5 mg/dL and 190 ± 8 mg/dL, respectively, and did not differ across experiments (all *p* ≥ 0.2). Subjects were sacrificed at three different time points to assess the persistent effects of AIE treatment on neurogenesis in the dorsal and ventral hippocampus (see Figure 1A). Subjects were sacrificed on P56 (24 h post-AIE treatment) to determine the acute effects of AIE treatment on neurogenesis in the late adolescent hippocampus. A separate group of subjects were sacrificed on P80 (25 days post-AIE treatment) to assess the effects of AIE on neurogenesis in the young adult hippocampus and to ensure that neurogenesis is persistently reduced following AIE treatment (see Broadwater et al., 2014). Finally, subjects were sacrificed on P220 (165 days post-AIE treatment) to assess the long-term, persistent effects of AIE treatment on neurogenesis in the adult hippocampus. For the duration of AIE exposure, subjects evidenced dramatic increases in body weight that did not differ as a function of treatment during AIE exposure (all *p*'s > 0.05, P25: CON = 73 ± 1 g, AIE = 73 ± 1 g; P55: CON = 304 ± 6 g, AIE = 286 ± 6 g; P80: 405 ± 8 g, AIE = 389 ± 7 g). Although body weights were unaffected by AIE treatment, there was an 11% (±3%) reduction in body weight of AIE-treated animals by P220 (CON = 622 ± 18 g, AIE = 553 ± 17 g [One-Way ANOVA: *F* = 7.9, *p* < 0.05]).

### Lipopolysaccharide (LPS) treatment

On P70, CON-, and AIE-treated animals received a single intraperitoneal injection of 1.0 mg/kg LPS (E. Coli, serotype 0111:B4; Sigma-Aldrich, St. Louis, MO) or saline. Subjects were monitored for 24 h following LPS administration for sickness behavior and sacrificed 10 days later on P80.

### Object recognition memory assessment

In the P220 sacrifice group, object recognition memory was assessed from P163 to P165 using an open-field apparatus. The open-field was constructed of wood (65 cm × 65 cm × 47 cm) and painted black with a white 4 × 4 grid painted on the base. The testing environment was illuminated by four 100-watt lights suspended above the apparatus, and white noise was provided by a white noise generator. An automated tracking system (Ethovision XT 8.0, Noldus Ethovision; Leesburg, VA) was used to monitor behavior, and between each subject the open-field and each object was thoroughly cleansed with Roccal-D Plus (Fisher Scientific; Pittsburgh, PA) to remove all olfactory cues. Before each trial, subjects were individually habituated to the testing environment for 10 min. On the first day of testing (habituation phase), individual rats were placed in the open-field in the bottom right facing the corner. The subjects were allowed to freely exposure the open-field for 5 min, and distance traveled (cm), latency to enter the center (s), and duration and entries into the center were recorded. Twenty-four hour later during the familiarization phase (Trial 2), two texturally and visually distinct objects (glass Mason jar [Object A] and plastic bottle [Object B]) were placed in opposite corners in the middle of the open-field apparatus. The subjects were allowed to freely exposure the open-field and objects for 5 min. Duration and number of contacts with each object was quantified as was the distance traveled (cm). Twenty-four hour later on the third day of testing (testing phase), Object B was replaced with novel Object C (ceramic mug), and the duration and number of contacts with the familiar (F) and novel (N) object was quantified as was the distance traveled (cm). A contact was defined as the subject directing the nose <2 cm from the object, or directly touching it. The discrimination ratio, which provides a measure of novel object exploration (Bartolini et al., 1996; Antunes and Biala, 2012), was calculated by: (Novel – Familiar)/(Novel + Familiar). The discrimination ratio varies between +1 and –1, with a positive ratio signifying more time spent exploring the novel object while a negative ratio reflects more time spent with the familiar object. A ratio of 0 indicates that equal time was spent exploring both objects (Antunes and Biala, 2012).

### Perfusion, brain tissue preparation, and immunohistochemistry

At the conclusion of each experiment, animals were anesthetized with a euthanization dose of sodium pentobarbital (100 mg/kg, i.p.) and transcardially perfused with 0.1 M phosphate-buffered saline (PBS, pH 7.4) followed by 4.0% paraformaldehyde in PBS. Brains were excised and post-fixed in 4.0% paraformaldehyde for 24 h at 4°C followed by 4 days of fixation in 30% sucrose solution. Coronal sections were cut (40 μm) on a sliding microtome (MICROM HM450; ThermoScientific, Austin, TX), and sections were sequentially collected into well plates and stored at –20°C in a cryoprotectant solution (30% glycol/30% ethylene glycol in PBS) for later immunohistochemistry.

Free-floating sections (every 12th section) were washed in 0.1 M PBS, incubated in 0.3% H_2_O_2_ to inhibit endogenous peroxidases, and blocked with normal serum (MP Biomedicals, Solon, OH). Sections were incubated in either goat polyclonal anti-doublecortin (DCX; Santa Cruz Biotechnology, Santa Cruz, CA), rabbit polyclonal anti-Ki-67 (Abcam, Cambridge, MA), or rabbit polyclonal anti-cleaved caspase-3 (Cell Signaling Technology, Danvers, MA) for 24 h at 4°C. Sections were then washed with PBS, incubated for 1 h in biotinylated secondary antibody (Vector Laboratories, Burlingame, CA), and incubated for 1 h in avidin-biotin complex solution (Vectastain ABC Kit; Vector Laboratories). The chromagen, nickel-enhanced diaminobenzidine (Sigma-Aldrich, St. Louis, MO), was used to visualize immunoreactivity. Tissue was mounted onto slides, dehydrated, and coverslipped. Negative control for non-specific binding was conducted on separate sections employing the abovementioned procedures with the exception that the primary antibody was omitted.

### Microscopic quantification and image analysis

Across studies, BioQuant Nova Advanced Image Analysis (R&M Biometric, Nashville, TN) was used for image capture and analysis. Images were captured using an Olympus BX50 microscope and Sony DXC-390 video camera linked to a computer, and all sections were quantified using an Olympus microscope equipped with an Olympus UPlan Fl objective (10X/0.30). For each measure, the microscope, camera, and software were background corrected and normalized to preset light levels to ensure fidelity of data acquisition. A modified stereological profile quantification method was used to quantify immunopositive cells within the hippocampal dentate gyrus as we have previously published that a comparison of unbiased stereological methodology with profile cell counting methods yielded nearly identical values relative to control subjects (Crews et al., 2004). Assessment of DCX was performed throughout the dorsal and ventral hippocampal dentate gyrus according to the atlas of Paxinos and Watson (1998 [see Figure 1B]). The total number of Ki-67 and cleaved caspase-3 immunoreactive cells were quantified throughout the dorsal hippocampus, and data are expressed as cells per mm^2^. We focused our assessment of Ki-67 and cleaved caspase-3 on P80 as it is long after the cessation of alcohol treatment, and the dorsal hippocampal dentate gyrus, which is the most commonly studied neurogenic region. Since DCX expression was densely distributed throughout subgranular zone of the hippocampal dentate gyrus making identification of individual neurons difficult, DCX + IR pixel density was rigorously thresholded to normalize pixel intensity (Vetreno et al., 2013). The threshold for pixel density was determined from the control subjects by calculating the average of the darkest and lightest values from each region of interest, and sections were imaged under identical conditions to avoid non-systematic variations (Beynon and Walker, 2012). Further, DCX + IR cell bodies were quantified in the superior blade of the dorsal hippocampal dentate gyrus of P80 animals using an Olympus UPlan Fl objective (40X/0.75) with a 2X magnifier, and compared to DCX pixel density. The outlined regions of interest were determined and staining density calculated by dividing the pixel count by the overall area (mm^2^).

### RNA extraction and reverse transcription polymerase chain reaction (RTPCR)

Hippocampal samples from the rat were collected according to the atlas of Paxinos and Watson (1998), and reverse transcribed as previously described (Vetreno and Crews, 2012; Vetreno et al., 2013). The primer sequences are presented in Table 1. Differences in primer expression between groups were expressed as cycle time (Ct) values normalized with β-actin, and relative differences between groups were calculated and expressed as the percent difference relative to CONs.

**Table 1:** **List of primers for RT-PCR**.

**Primer**	**Forward**	**Reverse**
RAGE	5′-AAC TAC CGA CTC CGA GTL TAC C-3′	5′-ACA ACT GTC CCT TTG CCA TCA-3′
TNFα	5′-ATG TGG AAC TGG CAG AGG AG-3′	5′-ACG AGC AGG AAT GAG AAG AAG-3′
Mac-1	5′-CTG CCT CAG GGA TCC GTA AAG-3′	5′-CCT CTG CCT CAG GAA TGA CAT C-3′
MCP1	5′-TCA CGC TTC TGG GCC TGT TG-3′	5′-CAG CCG ACT CAT TGG GAT CAT C-3′
CD14	5′-GAT CTG TCT GAC AAC CCT GAG T-3′	5′-GTG CTC CAG CCC AGT GAA AGA-3′
TLR4	5′-CCA GAG CCG TTG GTG TAT CT-3′	5′-TCA AGG CTT TTC CAT CCA AC-3′
HMGB1	5′-ATG GGC AAA GGA GAT CCT A-3′	5′-ATT CAT CAT CAT CAT CTT CT-3′
β1-Integrin	5′-GGC GGA CGC TGC GAA AAG AT-3′	5′-GAT ATG CGC TGC TGA CCA ACA AGT-3′
β3-Integrin	5′-AGA ACT CGC CCC GCT GTA ACC-3′	5′-CCC CGG GAT GAG CTC ACT GTA AT-3′
MMP-9	5′-AAG CCT TGG TGT GGC ACG AC-3′	5′-TTG AAA TAC GCA GGG TTT GC-3′
NF-κB (p65)	5′-CGA TCT GTT TCC CCT CAT CT-3′	5′-ATT GGG TGC GTC TTA GTG GT-3′
β-actin	5′-CTA CAA TGA GCT GCG TGT GGC-3′	5′-CAG GTC CAG ACG CAG GAT GGC-3′

### Statistical analysis

The Statistical Package for the Social Sciences (SPSS; Chicago, IL) was used for all statistical analyses. Analysis of variance (ANOVA) was used to assess BECs and body weights as well as the immunohistochemistry, RTPCR, and behavioral data. Pearson correlations (r) were used to assess the association between DCX + IR and behavioral performance on the object recognition memory task. *Post-hoc* analyses were performed when appropriate using Tukey's HSD. All values are reported as mean ± SEM, and significance was defined as *p* ≤ 0.05.

## Results

### Adolescent binge ethanol exposure persistently reduces neurogenesis in the dorsal and ventral hippocampus

**Figure 2:**
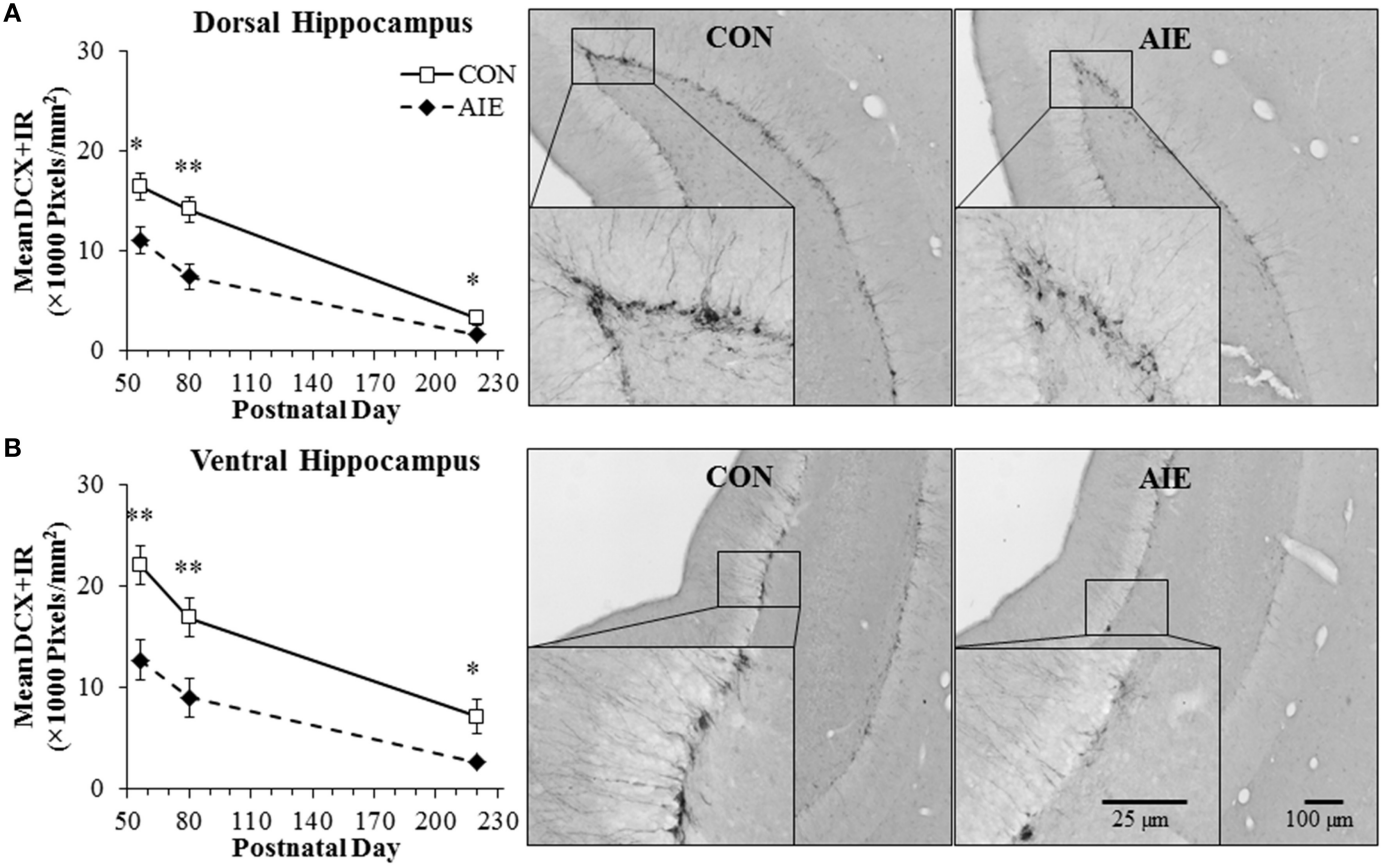
**Adolescent intermittent ethanol (AIE) exposure leads to long-term reductions of doublecortin-immunopositive (DCX + IR) cells in the dorsal and ventral hippocampal dentate gyrus. (A)** Quantification of pixel density revealed a 33% (±4%) decrease of DCX + IR in the adolescent (postnatal [P]56) dorsal hippocampal dentate gyrus that persisted from young adulthood (P80 [48 ± 7%]) into adulthood (P220 [51 ± 6%]), relative to controls (CONs). Included are representative photomicrographs of DCX + IR cells in the dentate gyrus of the dorsal hippocampus from young adult (P80) CON- and AIE-treated animals. **(B)** Quantification of pixel density revealed a 42% (±7%) decrease of DCX + IR in the adolescent (P56) ventral hippocampal dentate gyrus that persisted from young adulthood (P80 [46 ± 9%]) into adulthood (P220 [63 ± 7%]), relative to controls (CONs). Included are representative photomicrographs of DCX + IR cells in the dentate gyrus of the ventral hippocampus from young adult (P80) CON- and AIE-treated animals. Data are presented as mean ± SEM. ^*^indicates *p* < 0.05; ^**^indicates *p* < 0.01, relative to CON rats.

Doublecortin, a neuroprogenitor microtubule-associated protein expressed specifically by immature neurons (Brown et al., 2003), was assessed separately in the dorsal and ventral hippocampus following adolescent binge ethanol treatment. In our animal paradigm, human adolescent drinking is modeled using an intermittent administration schedule consistent with known patterns of heavy weekend binge drinking, but not daily drinking associated with alcoholism in adulthood. Tissue samples were collected from the dorsal and ventral hippocampus, and DCX + IR was assessed in animals sacrificed on P56 (24 h post-AIE treatment), P80 (25 days post-AIE treatment), and P220 (165 days post-AIE treatment). In CON- and AIE-treated subjects, DCX + IR in the dorsal and ventral hippocampus was characterized by darkly stained cell bodies and processes that innervated the granular cell layer of the hippocampal dentate gyrus, with less cell and process staining observed in the AIE-treated animals (see Figure 2). A 2 × 3 ANOVA (Treatment [CON vs. AIE] × Age [P56 vs. P80 vs. P220]) found that expression of DCX significantly declined with age from P55 to P220 in both the dorsal [main effect of Age: *F*_(2, 39)_ = 51.5, *p* < 0.01] and ventral [main effect of Age: *F*_(2, 39)_ = 25.1, *p* < 0.01] hippocampal dentate gyrus. Further, we found that relative to CONs, AIE treatment resulted in a 33% (±4%) decrease in DCX + IR in the adolescent (P56) dorsal hippocampal dentate gyrus that persisted from young adulthood (P80 [48 ± 7%]) into adulthood [P220 [51 ± 6%]; main effect of Treatment: *F*_(1, 39)_ = 24.7, *p* < 0.01; see Figure 2A]. Since DCX labels both cell bodies and processes of immature neurons, we assessed DCX + IR cell bodies in the superior blade of the dorsal dentate gyrus of the P80 age group to verify the AIE-induced DCX reductions. We chose the superior blade because there were less overlapping DCX + IR cells in this region allowing for more precise quantification. We found that AIE treatment reduced the number of DCX + IR cells by 45% (±4%), relative to the CONs [One-Way ANOVA: *F*_(1,15)_ = 56.3, *p* < 0.01]. Further, we found that DCX pixel density in the dorsal hippocampus was positively correlated with DCX cell counts (*r* = 0.78, *N* = 16, *p* < 0.01). Similar to the dorsal hippocampus, AIE treatment led to a 42% (±7%) decrease in DCX + IR in the adolescent (P56) ventral hippocampal dentate gyrus that persisted from young adulthood (P80 [46 ± 9%]) into adulthood (P220 [63 ± 7%]), relative to CONs [main effect of Treatment: *F*_(1,39)_ = 25.3, *p* < 0.01; see Figure 2B]. There were no interactions of Treatment × Age in either analysis. Thus, these data reveal that adolescent binge ethanol exposure reduces DCX expression in the dorsal and ventral hippocampal dentate gyrus of the adolescent brain (P56) that follows the age-related decline in neurogenesis with AIE-induced deficits persisting into adulthood (P220), although the age-associated decline in DCX + IR obscures the AIE-induced differences, particularly within the dorsal hippocampal dentate gyrus, where it is largely diminished by 220 days of age.

### Neural progenitor cell populations diminished in the young adult hippocampal dentate gyrus following adolescent binge ethanol exposure

**Figure 3:**
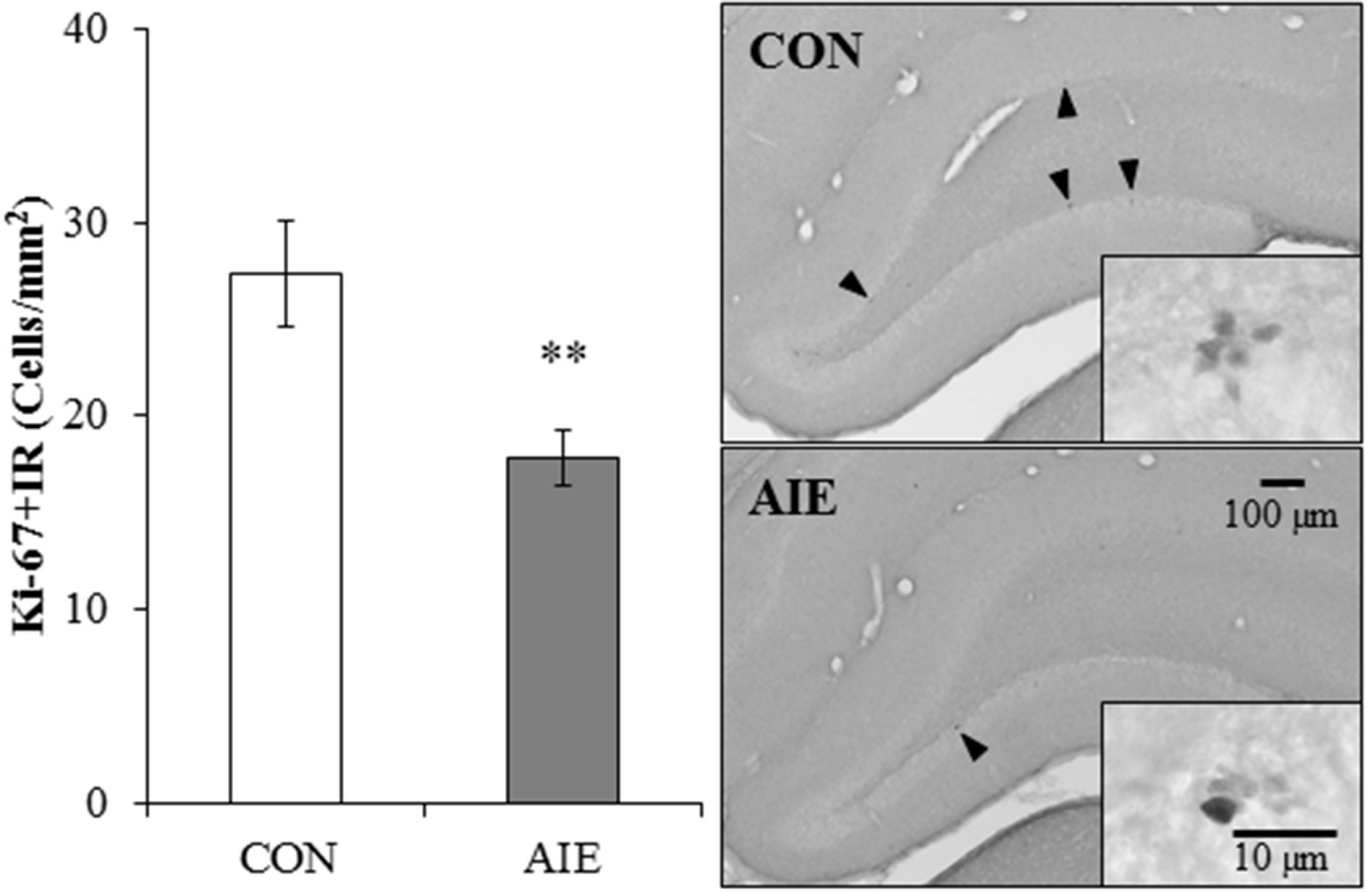
**Adolescent intermittent ethanol (AIE) treatment leads to long-term reductions of Ki-67 immunoreactive (+IR) expression in the young adult hippocampus**. Profile cell counts revealed a 35% (±5%) reduction of Ki-67 + IR cells in the dorsal hippocampal dentate gyrus of AIE-treated animals on postnatal day 80, relative to controls (CONs). Data are presented as mean ± SEM. ^**^indicates *p* < 0.01, relative to CON rats. Included are representative photomicrographs of Ki-67 + IR cells in the dentate gyrus of the dorsal hippocampus from CON- and AIE-treated animals. Arrowheads highlight Ki-67+IR cells.

Our finding of AIE-induced diminution of DCX + IR in the hippocampus prompted us to assess whether a reduction in neural progenitor cell proliferation may have contributed to the effects of adolescent binge ethanol exposure on neurogenesis. We assessed expression of Ki-67, an endogenous nuclear protein expressed in dividing cells (Scholzen and Gerdes, 2000), in the young adult (P80) dorsal hippocampal dentate gyrus of CON- and AIE-treated animals. In CON- and AIE-treated subjects, Ki-67 + IR was characterized by darkly stained clusters of cell bodies that were localized within the subgranular zone of the dentate gyrus. Adolescent binge ethanol exposure reduced Ki-67 + IR cell populations by 35% (±5%), relative to CON subjects [One-Way ANOVA: *F*_(1, 12)_ = 9.5, *p* < 0.01; see Figure 3]. These data suggest that diminished neural cell proliferation could contribute to the AIE-induced reduction in hippocampal neurogenesis.

### Cleaved caspase-3 immunoreactivity is significantly increased in the young adult hippocampal dentate gyrus following adolescent binge ethanol treatment

**Figure 4:**
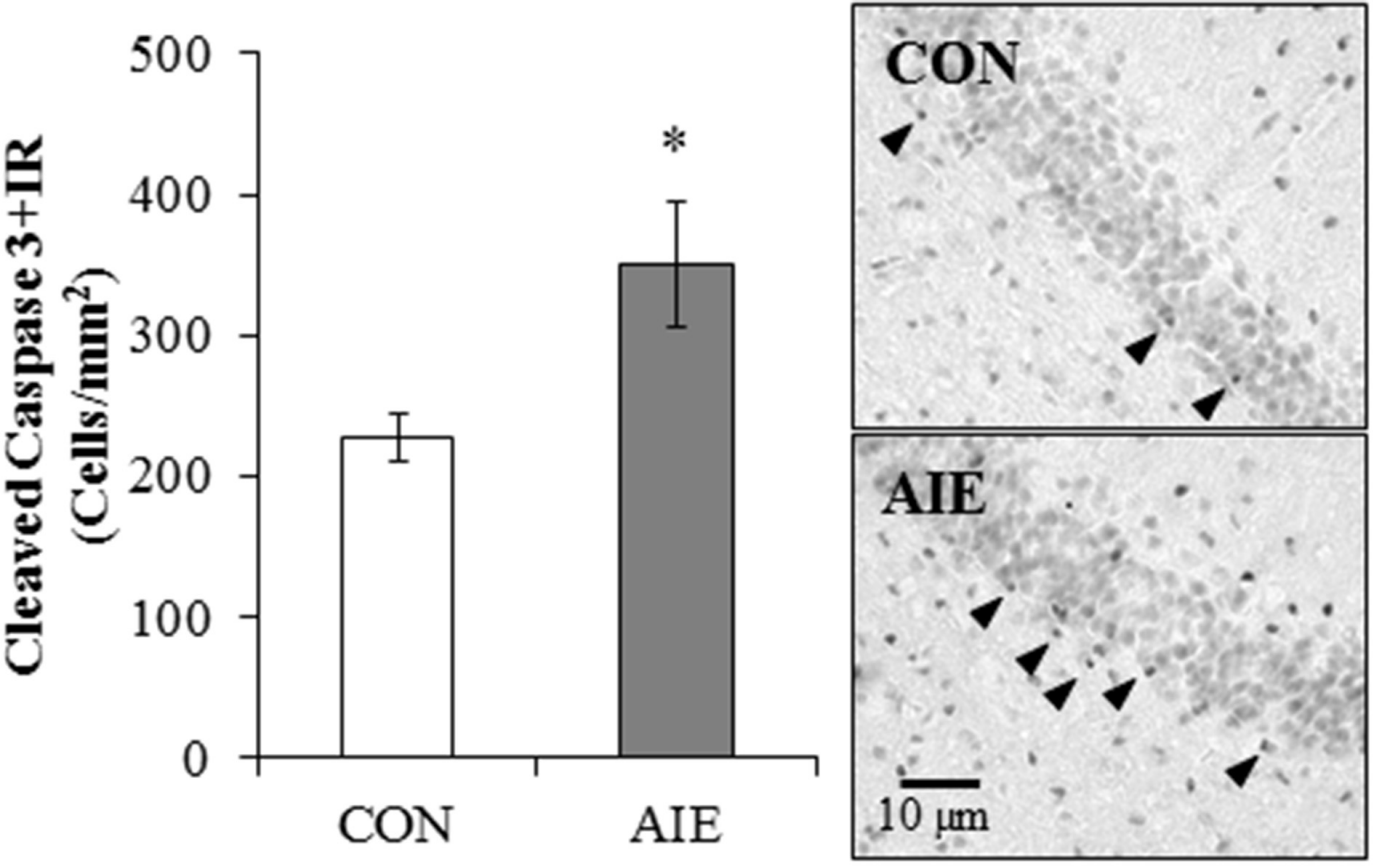
**Adolescent intermittent ethanol (AIE) treatment leads to long-term increased cleaved caspase-3 immunoreactive (+IR) expression in the young adult hippocampus**. Profile cell counts revealed a 54% (±20%) increase of caspase-3 + IR cells in the hippocampal dentate gyrus of AIE-treated animals on postnatal day 80, relative to controls (CONs). Data are presented as mean ± SEM. ^*^indicates *p* < 0.05, relative to CON rats. Included are representative photomicrographs of caspase-3 + IR cells in the dentate gyrus of the dorsal hippocampus from CON- and AIE-treated animals. Arrowheads highlight cleaved caspase-3+IR cells.

To determine if AIE treatment altered hippocampal cell death, we next assessed immunohistochemical expression of cleaved caspase-3, a marker of cellular death, in the young adult dorsal hippocampal dentate gyrus. Cleaved caspase-3 + IR in the CON- and AIE-treated animals was characterized by darkly stained cell bodies with increased staining evident in the AIE-treated animals. Adolescent binge ethanol treatment led to a significant 54% (±20%) increase in cleaved caspase-3 + IR cell populations, relative to CON subjects [One-Way ANOVA: *F*_(1, 12)_ = 6.4, *p* < 0.05; see Figure 4]. Thus, the increased neural cell death observed 25 days after the last ethanol treatment might contribute to the reductions of neurogenesis and/or cellular proliferation in the AIE-treated animals.

### Adolescent intermittent ethanol treatment leads to long-term upregulation of neuroimmune genes in the young adult hippocampus

Previous studies in adult rats have found that inhibition of NF-κB, a transcription factor known to increase proinflammatory gene expression, protects against ethanol-induced diminution of neurogenesis (Crews et al., 2006a). Further, in hippocampal-entorhinal cortex slice culture studies, ethanol increased neuroimmune gene expression with concomitant reductions of neurogenesis, an effect that was reversed with anti-inflammatory drug treatment (Zou and Crews, 2012). Since AIE treatment led to persistent reductions of neurogenesis and ethanol-induced upregulation of the neuroimmune system contributes to reductions of neurogenesis (Crews et al., 2006a; Zou and Crews, 2012), we assessed expression of proinflammatory cytokines, proteases, and other neuroimmune genes in the young adult hippocampus (P80) 25 days after the last binge ethanol exposure. Assessment of cytokine mRNA revealed an AIE-induced increase of TNFα [One-Way ANOVA: *F*_(1, 10)_ = 5.0, *p* < 0.05], MCP-1 [One-Way ANOVA: *F*_(1, 10)_ = 7.6, *p* < 0.05], and HMGB1 [One-Way ANOVA: *F*_(1, 10)_ = 5.6, *p* < 0.05], relative to CONs. Similarly, expression of the metalloproteinase MMP-9 was significantly increased in the hippocampus of AIE-treated animals in comparison to CON subjects [One-Way ANOVA: *F*_(1, 10)_ = 5.2, *p* < 0.05]. Measures of neuroimmune receptor mRNA revealed an approximate 2.5-fold increase in TLR4 [One-Way ANOVA: *F*_(1, 10)_ = 9.4, *p* < 0.05] as well as an approximate 3.5-fold increase in the TLR4 adaptor protein CD14 [One-Way ANOVA: *F*_(1, 10)_ = 9.0, *p* < 0.05]. Levels of RAGE, MAC-1, β1-integrin, and β3-integrin were unchanged in the young adult hippocampus following AIE exposure (all *p* > 0.1). Assessment of NF-κB (p65), a protein complex that regulates the neuroimmune response, found a 43% increase relative to CONs [One-Way ANOVA: *F*_(1, 10)_ = 5.1, *p* < 0.05; see Table 2]. These data reveal that AIE treatment leads to long-term upregulation of neuroimmune genes in the young adult hippocampus that might contribute to diminished hippocampal neurogenesis.

**Table 2:** **Adolescent intermittent ethanol (AIE) treatment increases mRNA expression of neuroimmune genes in the young adult hippocampus**.

	**mRNA (% of CON)**	
**Gene**	**CON**	**AIE**
β1-Integrin	100 ± 21	133 ± 14
β3-Integrin	100 ± 18	171 ± 46
***HMGB1***	***100 ± 13***	155 ± 19 ^*^
***MCP-1***	***100 ± 5***	226 ± 46 ^*^
***MMP-9***	***100 ± 8***	159 ± 25 ^*^
***TNF α***	***100 ± 9***	191 ± 39 ^*^
***NF-κB (p65)***	***100 ± 14***	143 ± 13 ^*^
***CD14***	***100 ± 14***	358 ± 85 ^*^
RAGE	100 ± 9	108 ± 9
***TLR4***	***100 ± 12***	266 ± 53 ^*^
Mac-1	100 ± 7	122 ± 12

RT-PCR assessment of neuroimmune signaling molecules in hippocampal tissue samples from postnatal day 80 rats revealed an approximate 55% increase of high-mobility group box 1 (HMGB1), an approximate 2-fold increase in monocyte chemoattractant protein-1 (MCP-1), a 59% increase in matrix metallopeptidase 9 (MMP-9), and a 91% increase in tumor necrosis factor alpha (TNFα), relative to CONs. Nuclear factor kappa-light-chain-enhancer of activated B cells (NF-κB) promoter 65 (p65) was increased by 43% in the AIE-treated animals, relative to CONs. Cluster of differentiation 14 (CD14), a Toll-like receptor 4 (TLR4) co-receptor, and TLR4 mRNA was increased by approximately 3.5-fold and 2.5-fold in AIE-treated animals relative to CONs, respectively. RT-PCR analyses were run in triplicate.

* *p < 0.05, relative to CONs. Data are presented as mean ± S.E.M*.

### Lipopolysaccharide exposure mimics adolescent binge ethanol-induced reductions of DCX + IR in the hippocampal dentate gyrus

**Figure 5:**
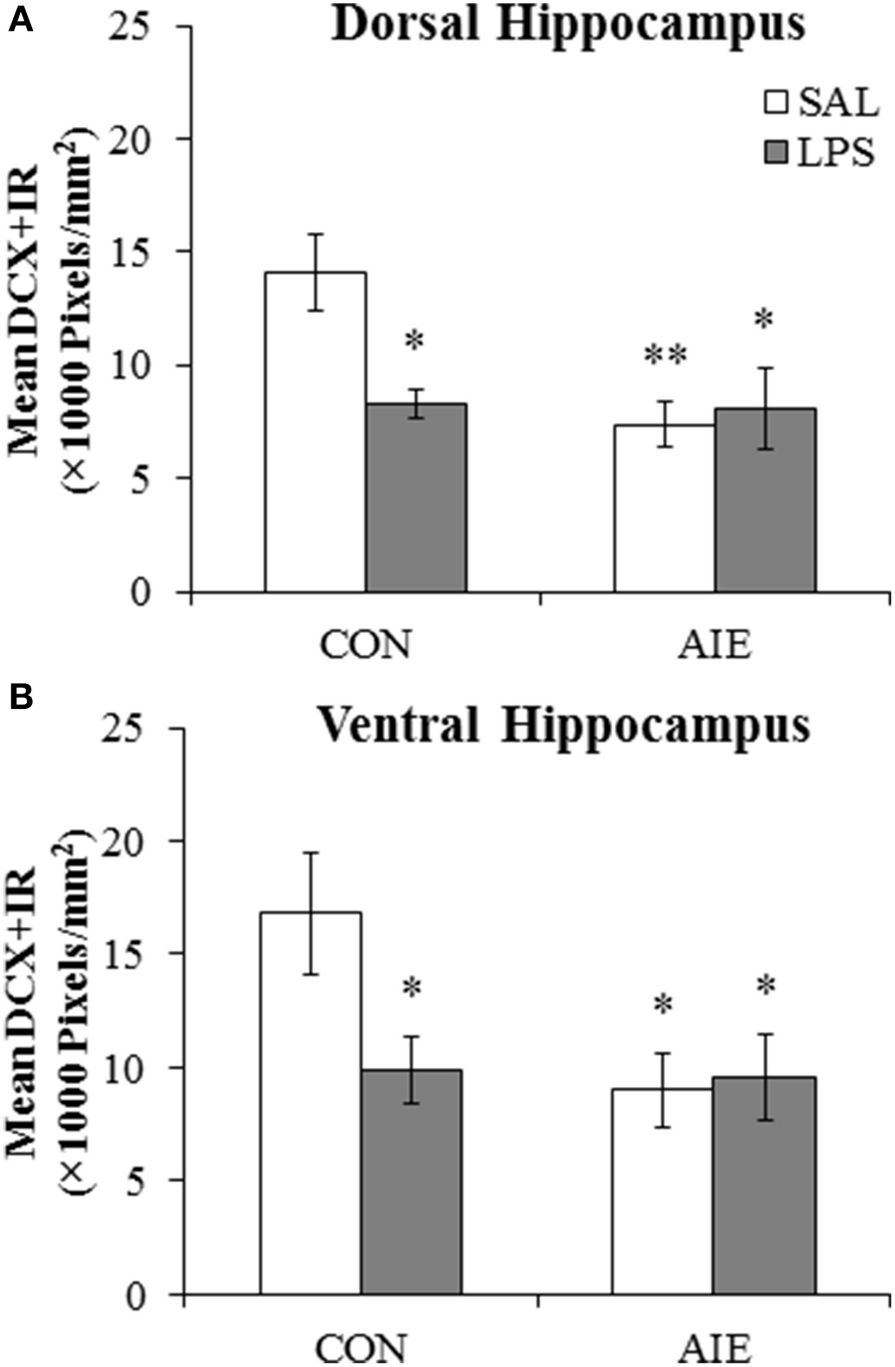
**Exposure to lipopolysaccharide (LPS) mimics the loss of doublecortin-immunoreactive (DCX + IR) cells associated with adolescent intermittent ethanol (AIE) exposure. (A)** Pixel density quantification in the dentate gyrus of the dorsal hippocampus of young adult rats (postnatal [P]80) revealed a significant reduction of DCX + IR in AIE- (48 ± 5%), CON + LPS (41 ± 7%), and AIE + LPS-exposed (42 ± 15%) rats, relative to CONs. **(B)** Pixel density quantification in the dentate gyrus of the ventral hippocampus of young adult rats (P80) revealed a significant reduction of DCX + IR in AIE- (46 ± 9%), CON + LPS (41 ± 9%), and AIE + LPS-exposed (41 ± 13%) rats, relative to CONs. ^*^ indicates *p* < 0.05 and ^**^ indicates *p* < 0.01, relative to CON/SAL rats. Data are presented as mean ± SEM.

Our laboratory previously found that adolescent binge ethanol exposure persistently upregulates neuroimmune signaling molecules and Toll-like receptor 4 (TLR4) expression in the brain (Vetreno and Crews, 2012; Vetreno et al., 2013). To determine if neuroimmune activation contributes to the reduction of hippocampal neurogenesis, CON- and AIE-exposed animals received a single dose of LPS (1.0 mg/kg, i.p.) on P70, and DCX + IR was assessed in the dorsal and ventral hippocampal dentate gyrus on P80. Lipopolysaccharide (LPS) is a gram-negative endotoxin agonist at TLR4 whose activation leads to proinflammatory cytokine and oxidase induction in brain (Qin et al., 2007). Expression of DCX in the dorsal and ventral hippocampal dentate gyrus was analyzed using separate 2 × 2 ANOVAs (Treatment [CON vs. AIE] × Drug [LPS vs. SAL]). Within the dorsal hippocampal dentate gyrus, AIE treatment significantly reduced DCX + IR relative to CONs [main effect of Treatment: *F*_(1, 28)_ = 6.2, *p* < 0.05]. Although there was no main effect of Drug (*p* > 0.05), there was a significant Treatment × Drug interaction [*F*_(1, 28)_ = 5.7, *p* < 0.05]. *Post-hoc* analysis revealed that, relative to CONs, DCX + IR was significantly reduced in the AIE (48% [±5%]; *p* < 0.01), CON + LPS (41% [±7%]; *p* < 0.05), and AIE + LPS (42% [±13%]; *p* < 0.05) treatment groups (see Figure 5A). Analysis of the ventral hippocampal dentate gyrus revealed a significant main effect of Treatment [*F*_(1, 28)_ = 4.3, *p* < 0.05; see Figure 5B]. There were no other main effects or interactions. Thus, these data reveal that LPS treatment induces a similar reduction of DCX + IR in the CONs that was observed in AIE-treated animals.

### Adolescent binge ethanol treatment induces long-term novel object recognition (NOR) memory impairments in adult rats

**Figure 6:**
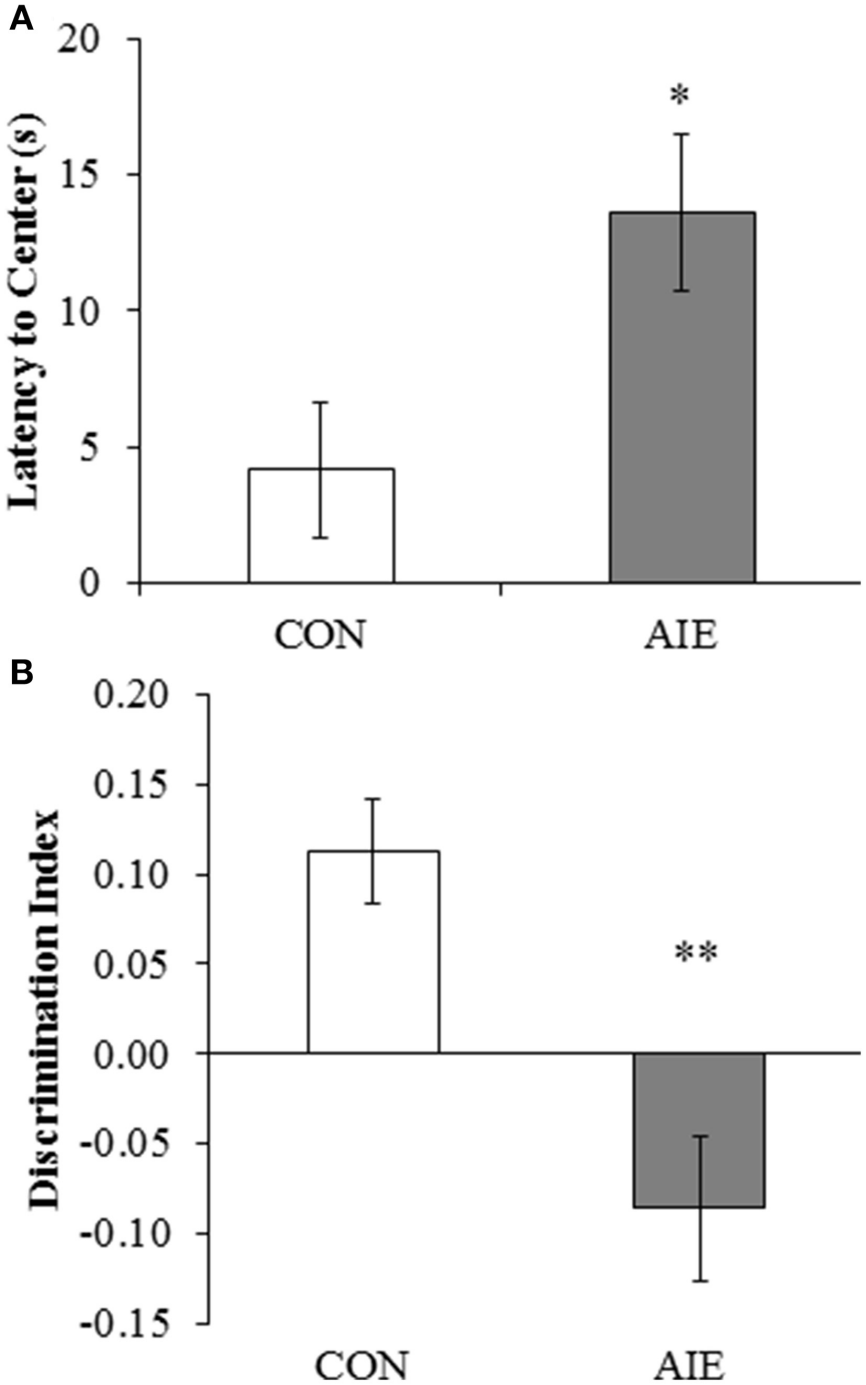
**Adolescent intermittent ethanol (AIE) treatment leads to long-term deficits in object recognition memory. (A)** Latency to enter the center of the open-field apparatus during the first trial, which provides a measure of thigmotaxis, was significantly increased in adult rats following AIE treatment. **(B)** Adolescent intermittent ethanol treatment significantly reduced the discrimination index, which is indicative of impaired object recognition memory, in adult rats relative to CONs. Data are presented as mean ± SEM. ^*^indicates *p* < 0.05; ^**^indicates *p* < 0.01, relative to CON rats.

Previous studies found that adolescent binge ethanol treatment of mice reduces object recognition memory 3 weeks after ethanol treatment (Pascual et al., 2007). Employing an open-field, we assessed NOR memory from P163 to P165 in adult rats (P220) following adolescent binge ethanol treatment. During the habituation phase, AIE-treated animals evidenced increased latencies to enter the center of the apparatus, relative to CONs [*F*_(1, 13)_ = 6.3, *p* < 0.05; see Figure 6A]. There was no effect of AIE treatment on distance traveled, duration in the center, or number of entries into the center (all *p* > 0.08). Adolescent binge ethanol exposure did not affect any of the measures during the familiarization phase (all *p* > 0.1). Further, neither group of subjects evidenced an object preference during the familiarization phase of the NOR task as evidenced by no difference in the number of contacts (both *p* > 0.7) or time spent in contact with either object (both *p* > 0.1). During the testing phase, AIE treatment significantly reduced the discrimination ratio relative to CONs [*F*_(1, 13)_ = 16.3, *p* < 0.01; see Figure 6B]. Together, these data reveal that AIE treatment leads to long-term impairments in hippocampal-dependent object recognition memory as well as increased latencies to enter the center of the maze.

### Novel object recognition memory and thigmotaxis is correlated with DCX + IR in the adult dorsal and ventral hippocampal dentate gyrus

**Figure 7:**
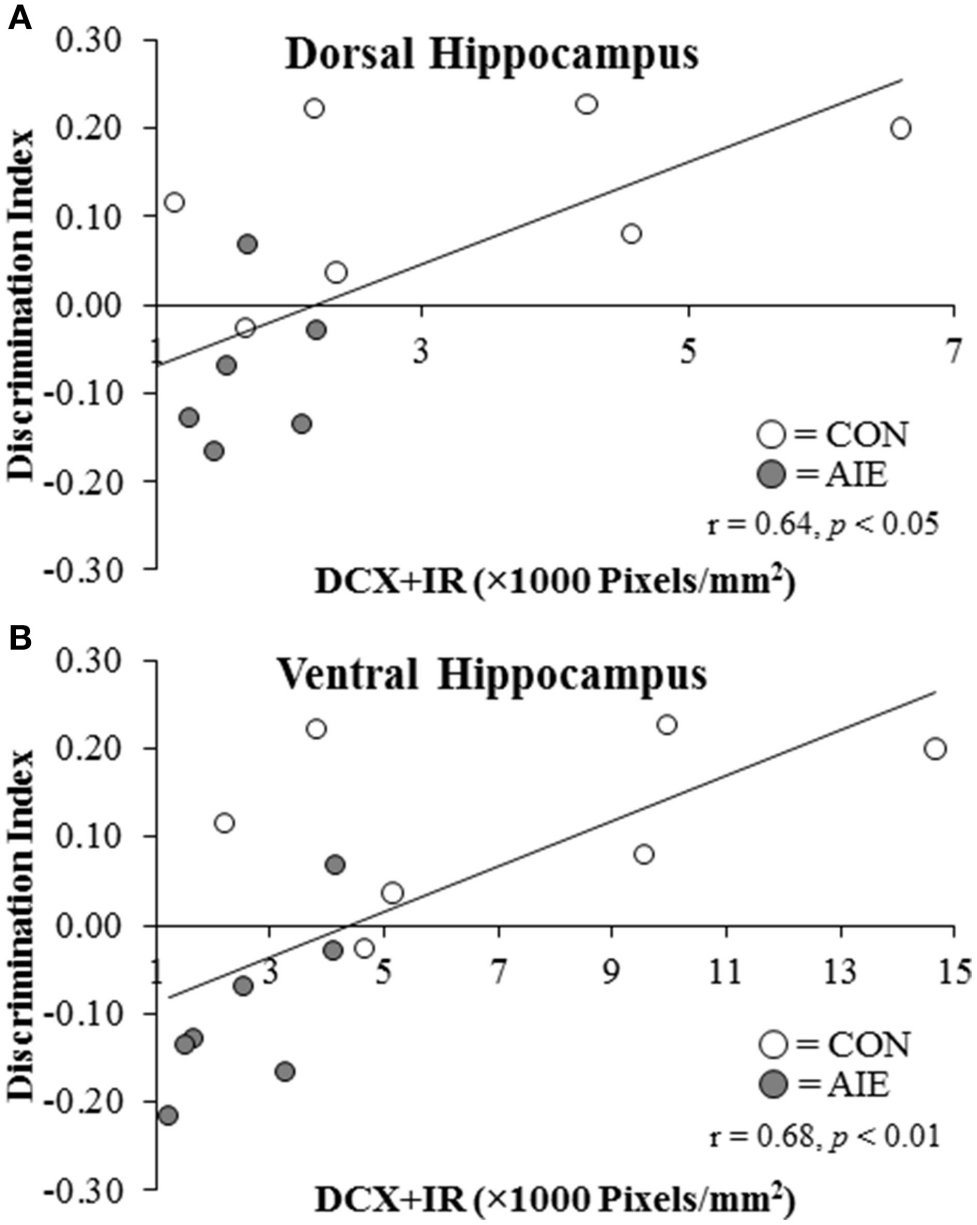
**Doublecortin immunoreactivity (DCX + IR) in the dorsal and ventral hippocampal dentate gyrus of P220 adult rats is correlated with object recognition memory performance**. Performance on the open-field object recognition memory test is positively correlated with DCX + IR in the **(A)** dorsal hippocampus (*r* = 0.64, *N* = 14, *p* < 0.01) and **(B)** ventral hippocampus (*r* = 0.68, *N* = 14, *p* < 0.01).

Since AIE treatment leads to persistent changes in hippocampal neurogenesis and neurogenesis is implicated in object recognition memory (Jessberger et al., 2009; Suarez-Pereira et al., 2015), we correlated the discrimination ratio obtained from the NOR test in the P220 subject group with expression of DCX in the dorsal and ventral hippocampal dentate gyrus of adult rats (P220). As depicted in Figure 7, the discrimination ratio was positively correlated with DCX + IR in both the dorsal (*r* = 0.64, *N* = 14, *p* < 0.05) and ventral (*r* = 0.68, *N* = 14, *p* < 0.01) hippocampal dentate gyrus of adult rats (P220). Given the involvement of the ventral hippocampus in anxiety-like behavior (McHugh et al., 2004), we next assessed the association between latency to enter the center of the open-field during the habituation phase, and expression of DCX in the dorsal and ventral hippocampal dentate gyrus of adult rats (P220). We found that DCX + IR in the ventral, but not dorsal, hippocampal dentate gyrus was negatively correlated with latency to enter the center of the apparatus (*r* = –0.54, *p* < 0.05, *N* = 14). Thus, hippocampal neurogenesis is correlated with both novel object recognition memory and anxiety-like behavior in the adult rats.

## Discussion

We report here for the first time that adolescent binge ethanol treatment leads to long-term reductions of DCX-immunopositive neurons in both the dorsal and ventral hippocampal dentate gyrus that persists from late adolescence (P56) into adulthood (P220). The attenuation of neurogenesis in the AIE-treated animals was accompanied by a corresponding reduction in the expression of the endogenous neural progenitor cell marker Ki-67 as well as an increase in the cell death marker, cleaved caspase-3. Adolescent binge ethanol treatment also led to a long-term upregulation of TLR4 and its endogenous agonist HMGB1 as well as other neuroimmune genes in the young adult hippocampus 25-days following the conclusion of binge ethanol exposure. Administration of the TLR4 agonist LPS induced a similar reduction of DCX + IR in the young adult dorsal and ventral hippocampal dentate gyrus of control animals that was observed following AIE treatment. Behaviorally, AIE treatment significantly increased thigmotaxia and impaired object recognition memory on the NOR memory task in the adult rat, a measure that is dependent on functionally intact hippocampal circuitry. Further, object recognition memory was positively correlated with DCX expression in both the dorsal and ventral dentate gyrus while thigmotaxia was negatively correlated with DCX + IR in the ventral hippocampus. In humans, alterations in hippocampal neurogenesis are observed in neurodegenerative disorders (for review, see Sierra et al., 2011), which likely contribute to the memory impairments, and perhaps psychological ailments (e.g., depression and anxiety), that accompany these conditions. Additionally, the long-term upregulation of neuroimmune genes in the hippocampus might contribute to binge ethanol-induced reductions of neurogenesis and facilitate hippocampal neurodegeneration later in life. Although we have not established a direct cause and effect of neuroimmune gene induction on neurogenesis in our AIE model, Zou and colleagues (Zou and Crews, 2012) found that ethanol-induced upregulation of neuroimmune signals in *ex vivo* slice culture reduced neurogenesis. Further, *in vivo* blockade of neuroimmune gene induction prevented ethanol-induced reductions of neurogenesis (Crews et al., 2006a). Taken together, these data support the hypothesis that adolescent binge ethanol exposure persistently reduces neurogenesis, potentially through a neuroimmune mechanism, leading to long-term impairments in hippocampal function.

Neurogenesis is a dynamic process that is highly susceptible to modulation by environmental influences. In adult rats, binge ethanol treatment does not induce long-term reductions of neurogenesis in the dorsal hippocampus (Broadwater et al., 2014). However, neurogenesis in the adolescent brain is uniquely vulnerable to the neurotoxic effects of ethanol. In the present study, we found that AIE treatment led to long-term reductions of hippocampal neurogenesis in both the dorsal and ventral dentate gyrus that persisted from P56 (late adolescence) into adulthood (P220). These findings are in agreement with previously published studies from our laboratory and others that reported adolescent binge ethanol-induced reductions of neurogenesis in the young adult dorsal dentate gyrus (Crews et al., 2006b; Ehlers et al., 2013; Broadwater et al., 2014). The reduction of DCX + IR in the present study was accompanied by decreased expression of Ki-67, an endogenous marker of neural progenitor cells. Diminished expression of Ki-67 has also been reported in rodent and non-human primate models of adolescent binge ethanol exposure (Taffe et al., 2010; Ehlers et al., 2013). While the mechanisms underlying the AIE-induced loss of neurogenesis are still unclear, it could involve an increase in neural cell death. In the present study, we found that AIE treatment led to a long-term increase in the expression of cleaved caspase-3. Ehlers et al. (2013) similarly reported that AIE treatment not only increased the expression of cleaved caspase-3, but also increased expression of the cell death marker, FluoroJade. Further, adolescent binge ethanol treatment of non-human primates led to persistent reductions of NeuroD and PSA-NCAM in the hippocampal dentate gyrus that was accompanied by increased FluoroJade + IR (Taffe et al., 2010). Together, these data reveal that adolescent binge ethanol treatment leads to persistent long-term reductions of neurogenesis in the dorsal and ventral hippocampal dentate gyrus that might be a consequence of diminished cellular proliferation and/or increased cell death.

Although the loss of hippocampal neurogenesis is consistently observed in animal models of adolescent binge ethanol exposure, the mechanism underlying this reduction is unclear. A recent *ex vivo* hippocampal-entorhinal cortex (HEC) slice culture study from our laboratory implicated the neuroimmune system in contributing to the deleterious effects of alcohol on neurogenesis. Treatment of HEC slices with ethanol (100 mM) for 4 days significantly reduced the expression of 5-bromo-2-deoxyuridine (an S-phase cell cycle mitotic marker), Ki-67, and DCX that was accompanied by an increase in cytokine and inflammasome complex expression (Zou and Crews, 2012). Further, treatment of these HEC slices with either IL-1β or alcohol in combination with anti-inflammatory drugs led to concomitant reductions and increases in DCX + IR, respectively. In the present study, AIE treatment led to the long-term upregulation of proinflammatory cytokines (TNFα, MCP-1, and HMGB1) and proteases (MMP-9) as well as NF-κB (p65), which is a protein complex that modulates the neuroimmune response. NF-κB (p65) is essential for NF-κB nuclear translocation and induction of proinflammatory cytokines. Its activation leads to further NF-κB synthesis providing evidence for the establishment of positive loops of neuroimmune activation (Crews et al., 2011), and likely contributes to the persistent neuroimmune gene induction observed in the hippocampus. In addition, mRNA expression of the HMGB1 receptor TLR4 and its adaptor protein CD14 were also upregulated in the young adult hippocampus 25-days following the conclusion of AIE treatment. While the contributions of these neuroimmune molecules to the AIE-induced reductions of neurogenesis are still a matter of debate, Rolls et al. (2007) found that genetic knockout of TLR4 increased neurogenesis in transgenic mice. Furthermore, application of recombinant IL-6 and TNFα to *in vitro* hippocampal precursor cells reduced neurogenesis (Monje et al., 2003). Another possible mechanism contributing the AIE-induced reductions of neurogenesis involves the cholinergic system of the basal forebrain. Cholinergic neurons of the basal forebrain project to newborn neurons and modulate neurogenesis in the hippocampal dentate gyrus (Mohapel et al., 2005; Kotani et al., 2006). Interestingly, acetylcholine also regulates inflammation through the alpha 7 nicotinic receptor (Wang et al., 2003), and AIE treatment was found to persistently reduce cholinergic marker expression in the adult brain (Vetreno et al., 2014). Together, these data support the hypothesis that activation of the neuroimmune system in the hippocampus might contribute to the persistent AIE-induced loss of neurogenesis. Indeed, we have previous reported that AIE treatment leads to long-term upregulation of neuroimmune genes in the young adult brain (Vetreno and Crews, 2012; Vetreno et al., 2013). Interestingly, administration of LPS, which activates TLR4 leading to the induction of proinflammatory neuroimmune genes, produced a similar reduction of neurogenesis in control animals that was observed following AIE treatment. Although we did not directly show a cause and effect relationship in between neuroimmune gene induction and neurogenesis in the present study, our findings of a long-term upregulation of neuroimmune genes in the hippocampus, coupled with previously published studies supporting a role for neuroimmune gene induction in reduction of neurogenesis (Monje et al., 2003; Crews et al., 2006a; Rolls et al., 2007; Zou and Crews, 2012), support the hypothesis that induction of neuroimmune genes contributes to reductions of neurogenesis. Thus, these data support a role for ethanol-induced activation of the innate immune system in contributing to the persistent loss of hippocampal neurogenesis.

Hippocampal neurogenesis has been implicated in hippocampal-mediated cognitive function. There is a reciprocal interaction of neurogenesis with learning and memory as performance on hippocampal-dependent learning and memory tasks facilitate the neurogenic process (Gould et al., 1999a) while neurogenesis is critically involved in hippocampal-dependent learning and the establishment of memory (Madsen et al., 2003). Similarly, the ventral hippocampus and neurogenesis have also been implicated in modulating emotive functioning (McHugh et al., 2004; Revest et al., 2009). In the present study, we found that AIE treatment impaired hippocampal-dependent novel object recognition memory performance in adulthood, an effect that was positively correlated with expression of DCX in both the dorsal and ventral hippocampal dentate gyrus. The relationship between neurogenesis and novel object recognition memory is uncertain with some studies indicating a relationship (Jessberger et al., 2009; Suarez-Pereira et al., 2015) and others not (Aggleton and Brown, 2005). In the present study, we found that AIE treatment impaired hippocampal-dependent novel object recognition memory performance in adulthood, an effect that was positively correlated with expression of DCX in both the dorsal and ventral hippocampal dentate gyrus. Although the exact role of neurogenesis is unknown, reductions in neurogenesis in a large number of studies is associated with an increased risk for the development of psychopathology and cognitive dysfunction. We also found that neurogenesis in the ventral hippocampus was negatively correlated with thigmotaxia, which provides a measure of anxiety-like behavior (Treit and Fundytus, 1988). Although a number of hypotheses have been advanced regarding the contributions of neurogenesis to hippocampal functioning, a recently published study asserts that neurogenesis is critical for the refinement and maintenance of neural circuitry. Sensory deprivation-induced reductions of neurogenesis in the olfactory bulbs resulted in a broadening of intrabulbar projections, indicating that neurogenesis is critical for maintaining circuit specificity. Indeed, reinstatement of sensory inputs recovered neurogenesis leading to increased specificity of intrabulbar projections (Cummings et al., 2014). Although the previously mentioned study focused on neurogenesis in the olfactory bulbs, it is plausible that neurogenesis in the hippocampus plays a similar role in the refinement and maintenance of hippocampal circuitry, and that its disruption by AIE treatment leads to cognitive and emotive dysfunction.

Age-associated reductions of hippocampal neurogenesis have been reported in both human (Spalding et al., 2013) and animal studies (Kuhn et al., 1996), and likely contribute to the cognitive decline commonly observed in the aging population (Van Praag et al., 2005). We found that DCX + IR in the dorsal and ventral hippocampal dentate gyrus of CON- and AIE-treated animals decreased significantly from late adolescence (P56) into adulthood (P220). Broadwater et al. (2014) reported a similar progressive decline of neurogenesis in the dorsal dentate gyrus from P74 to P116. The age-associated reductions of hippocampal neurogenesis likely contribute to the cognitive decline and increased incidence of depression and other psychopathologies that are associated with pathological aging. Indeed, although the onset of depression and other psychological disorders are uncommon in old age, diminished plasticity and neurogenesis early in life might predispose an aging individual to the etiological development of psychopathology, such as depression (Klempin and Kempermann, 2007). Thus, adolescent binge drinking-induced diminution of hippocampal neurogenesis and plasticity might increase the likelihood of the development of psychopathology later in life.

In conclusion, adolescent binge ethanol exposure persistently reduces hippocampal neurogenesis from late adolescence into adulthood in both the dorsal and ventral hippocampal dentate gyrus. This reduction in neurogenesis was accompanied by increased cell death (i.e., cleaved caspase-3 + IR cells) as well as long-term upregulation of TLR4, HMGB1, and other neuroimmune signaling molecules. Although the mechanism underlying this reduction remains to be fully elucidated, the LPS data support the hypothesis that AIE-induced innate immune gene induction might contribute to the persistent loss of hippocampal neurogenesis. Adolescent binge ethanol treatment led to long-term deficits in hippocampal-dependent novel object recognition memory and increased anxiety-like behavior, both of which were correlated with DCX expression in the dorsal and ventral hippocampus. These novel findings reveal that an early life insult (i.e., adolescent binge drinking) impart long-term changes to the brain that contribute to cognitive and emotive dysfunction later in life.

### Conflict of interest statement

The authors declare that the research was conducted in the absence of any commercial or financial relationships that could be construed as a potential conflict of interest.
